# Cell culture, sex determination and single cell cloning of *ovine* transgenic satellite cells *in vitro*

**DOI:** 10.1186/s40709-014-0022-z

**Published:** 2014-12-24

**Authors:** Fatemeh Salabi, Mahmood Nazari, Wen G Cao

**Affiliations:** Transgenic and Stem Cell Core, Institute of Animal Science and Veterinary Medicine, Chinese Academy of Agricultural Sciences, Beijing, 100193 People’s Republic of China; Animal Science Department, Khuzestan Ramin Agriculture and Natural Resources, Khuzestan, Iran

**Keywords:** Satellite cells, Sex determination, Enzymatic disaggregation, Single cell cloning

## Abstract

**Background:**

This study was performed to describe the basic methods to isolate and culture of primary satellite cells (PSCs) obtained from 50 to 60-day-old sheep fetuses, single cell cloning of transfected PSCs and sexing of *ovine* PSCs based on the ZFY/ZFX, amelogenin and high-motility-group (HMG) box sequences.

**Results:**

Three-step enzymatic digestion method increased PSCs isolation from tissue and reduced the damage of cells during long time incubation with enzymes. The results of cloning showed that the 103 and 81 clones (from a total of 184 clones) were derived from feeder and bFGF treatment, respectively. The overall sexing efficiency in the present study was 100%. Southern blot results of sex determination were in complete agreement with PCR-amplified bands which confirmed that the HMG box of SRY gene amplified from the *ovine* genome and that was specific for male.

**Conclusions:**

We successfully isolated and cultured sheep primary satellite cells via mechanical and enzymatic disaggregation. Our finding demonstrated that use of feeder and addition of bFGF to the culture medium improved cloning efficiency. The results of sex detection demonstrated that these methods can be applied to detect the sex of primary satellite cells and to determine the sex of sheep embryo prior to produce sheep embryos by somatic cell nuclear transfer technique *in vitro*. Nevertheless, our findings suggested that sex determination of satellite cells base on amelogenin sequence can be accurate, relatively simple, rapid, and inexpensive.

## Background

Satellite cells are a population of adult muscle stem cells that play a key role in mediating muscle regeneration. These mononuclear cells are easy to obtain from *in vitro* culture, can be isolated with little harm to the structure and function of the tissues and organs and have strong proliferation capacities [1]. Also, satellite cells provide a stable model for tissue engineering studies, such as those involving the transplantation of muscle-derived satellite cells for muscle tissue reconstruction [2]. Furthermore, the established muscle-derived satellite cells model can also be used to study the genes associated with muscle development, and as seed cells for animal biotechnology-related studies. Most muscle-derived satellite cells studies have involved mice, rats and humans; in contrast, muscle-derived satellite cells studies are rare in livestock, such as cows and sheep.

Recent studies have showed that fetal skeletal muscle satellite cells have a flexible potential to be used for transgenic animal production by somatic cell nuclear transfer technique because these cells are muscle-derived stem cells that can potentially proliferate and differentiate. Since the single cell cloning became the obstacle of producing gene targeting clone, we tried to derive the transgenic cell lines from *ovine* satellite cells transfected with pEGFP-N1 plasmid as a model of transgenic satellite cell. In addition, sex identification for the pre-implanting embryo plays a very important role in commercial husbandry production. Several protocols have been established for sexing the embryos and cell lines in farm animals. Among of these methods, PCR-based sexing assays are generally favored, because of the advantages of being relatively simple, rapid, and inexpensive [3,4]. The key point of *ovine* sex determination by PCR is to design primers that are specific for rams and with high sensitivity, because the accuracy of sex determination is influenced by the primers. Reported primers for sex determination were derived from Y-chromosome repeat sequences [5], the amelogenin (AMEL) gene sequence [6], ZFY/ZFX gene sequences [7] and the SRY gene core sequence [8,9]. Prior to utilization of fetal transgenic satellite cells for nuclear transfer, sex detection of transgenic cell lines isolated from single cell cloning is necessary because the gender of transgenic embryo can be determined by sex detection of nuclear donor cells. Therefore, we investigated *in vitro* culture and cell cloning of sheep satellite cells to establish a sheep cell line and to develop an *ovine* primary satellite cells sexing assay that was accurate, inexpensive and relatively fast. The future goal is to apply these cells for the production of transgenic sheep by somatic cell nuclear transfer technique. Our findings provide an experimental basis for the research and application of satellite cells in other fields, such as livestock breeding.

## Results

### Culture of sheep primary satellite cells

**Figure 1:**
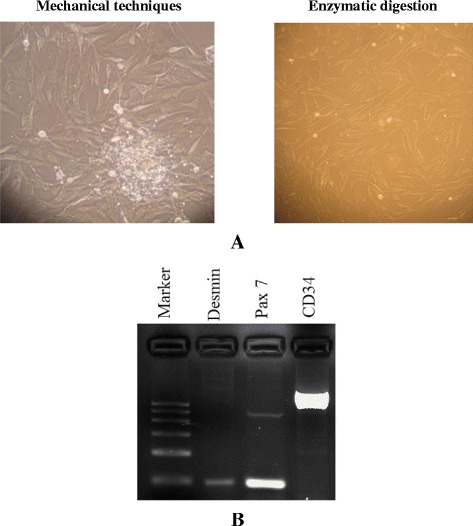
**Primary cultures and identification of PSCs derived from mechanical and enzymatic disaggregation. (A)** Enzymes treatment yielded the highest number of cells compared with mechanical disaggregation. **(B)** Desmin, Pax7 and CD34 were amplified with primers designed to produce an 101, 106 and 858-bp product in the primary satellite cells, respectively. Marker is 600 bp DNA ladder.

To investigate and develop an efficient method to isolate *ovine* primary satellite cells, collected muscle tissues were digested in three steps by two different enzymes of collagenase for 30 min, trypsin for 30 min followed by digestion with collagenase for 30 min again to induce muscle tissue digestion, and grown in DMEM with 20% FBS and 10% Hours serum. When the same amounts of muscle tissues were used, enzymes treatment was shown to yield the highest number of cells (Figure 1A) compared with mechanical disaggregation.

Cells were observed growing from sheep skeletal muscle within 1 week and 2 days for mechanical and enzymatic isolation, respectively. Primary cultures of PSCs derived from mechanical and enzymatic disaggregation grew to confluence in approximately 4 and 2 weeks, respectively (Figure 1A). Before utilizing these cells for single cell cloning and sex detection, we attempted to demonstrate that the cells were satellite cells. Therefore, PCR reactions were performed with Pax7, CD34 (satellite cell-specific markers) and Desmin (muscle cells-specific markers) primers. The results show that RT-PCR reactions indeed contained Pax7, Desmin and CD34 cDNA (Figure 1B).

### Analysis of clones derived from single cell cloning

**Figure 2:**
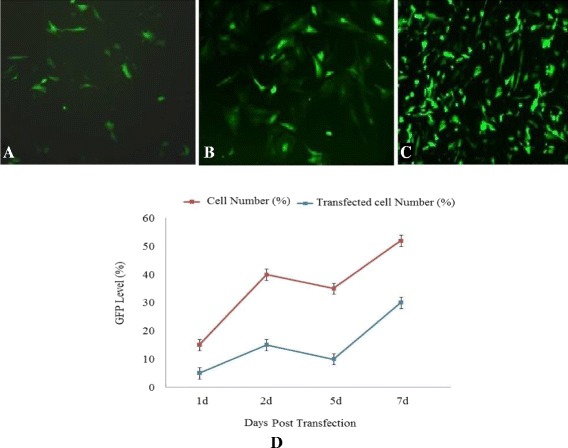
**Analysis of GFP expression in primary satellite cells transfected by pEGFP-N1 plasmid.** GFP fluorescence in PSCs transfected by pEGFP-N1 plasmid as visualized by fluorescent microscopy. **(A)** Transfected *ovine* fetus PSCs 24 hrs, **(B)** 48 hrs after first transfection **(C)** and 48 hrs after second transfection. **(D)** FACS analysis of GFP expression level in a time lapse. The y-axis shows mean of the expression levels and x-axis shows days after transfection. EGFP expression levels for transfected cells and cells number were quantified by measuring the extracellular EGFP by a fluorescence detector for 24 hrs and 48 hrs of first transfection, 3 days post-first transfection and 48 hrs after second transfection on days 1, 2, 5 and 7, respectively. FACS Analysis of GFP expression level for cell number showed that the percentage of transfected cells increased as increasing the transfection time.

FACS analysis of GFP expression was performed by a cell-based green fluorescent protein (GFP) reporter assay to determine the percentage of GFP-positive cells in each well for characterizing the transfection efficiency and using the GFP reporter system to carry out the cell sorting. At days 1 and 2 of first transfection following by 3 days post double transfection, GFP expression in PSCs was photographed under a fluorescent microscope (Figures 2A-C). Days 1, 2, 5 and 7 of transfection, the transfected pool was tested for GFP expression by flow cytometry and sorted based on the top 0.5% of GFP expression, collected and expanded. The results showed that percentage of transfected cells increased in intensity as the time of transfection with pEGFP-N1 increased. Double transfected cells showed a highest GFP expression level. This result confirmed that the efficiency of transfection was increased by increasing the incubation time and transfection time (Figure 2D).

The single-cell clones were derived from satellite cells transfected with pEGFP-N1 vector. It may be a potential screening system for getting target cell production. The transfected cells grew slowly in the first 2 weeks after single cell cloning, but they grew faster after re-stimulation the clones in group 2 or using feeder cells in group 3, then most of cells reached 30% confluency within 5–7 days. The results showed that the cloning efficiency was increased by using feeder cells and bFGF. A total of 184 clones were grown from total of 864 clones. The results demonstrated that 103 and 81 clones from all clones were obtained from bFGF and feeder derived clones, respectively (Table 1).

**Table 1:** **Summary of colonies derived from single cell cloning**

**Treatments**	**No. of group**	**Total of single cell cloned**	**Total of clones derived from single cell cloning**
Simple cloning	1	6 × 48	0
Feeder derived clones	2	6 × 48	81
bFGF derived clones	3	6 × 48	103
Total		864	184

### PCR amplification

#### HMG box

**Figure 3:**
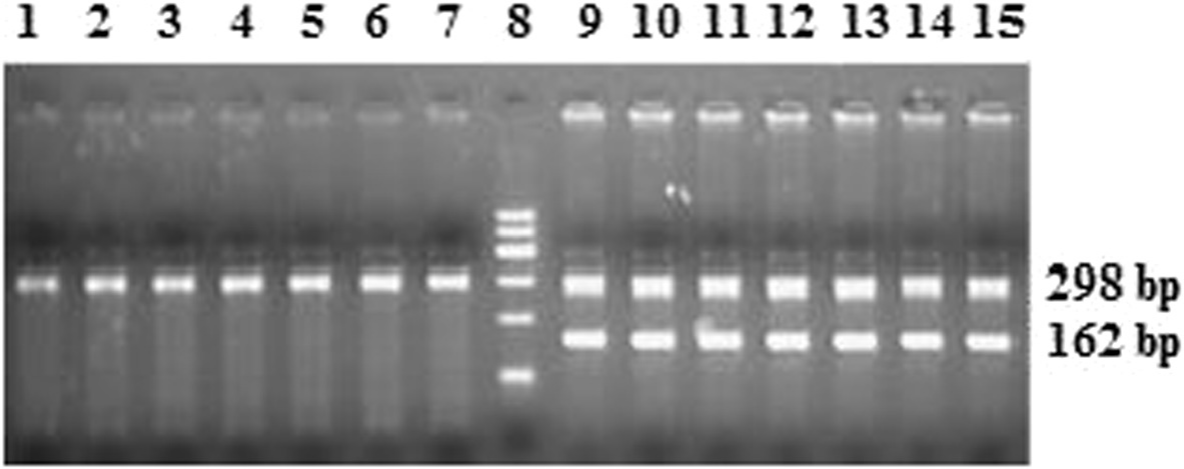
**Agarose gel electrophoresis of the duplex PCR products, using HMG-box primers and beta-acting primers.** The genomic DNA was extracted from male and female sheep. Lanes 1–7 are PCR products of genomic DNA from seven female sheep; lanes 9–15 are PCR products of genomic DNA from seven male sheep; lane 8 is 600 bp DNA ladder. The position of the 162 and 298 bp are the HMG-box product and beta-actin product, respectively.

The duplex PCR performed on genomic DNA from male and female sheep using the PCR parameters, HMG-box and beta-acting primers given in Table 2. As shown in Figure 3, we observed both 298 and 162 bp amplified fragments in male sheep but the 162 bp bands were not found in the female sheep. The results showed that no PCR products were obtained in female genetic DNA using the HMG-box primer and the HMG-box of sheep SRY gene was male-specific sequence.

**Table 2:** **Primer sequences, PCR conditions and PCR product sizes of ZFX/ZFY, HMG and amelogenin (AMEL) loci**

**Gene**	**Primer sequence**	**T (°C)** ^**a**^	**Product size (bp)**	
			**Male**	**Female**
ZFX/Y^b^				
Forward	5΄-ATAATCACATGGAGAGCCACAAGCT-3΄	58	445	445
Reverse	5΄-GCACTTCTTTGGTATCTGAGAAAGT-3΄	58		
HMG box of SRY gene^c^				
Forward	5΄-TGAACGCTTTCATTGTGTGGTC-3΄	58	162	-
Reverse	5΄-GCCAGTAGTCTCTGTGCCTCCT-3΄	58		
Beta-actin^c^				
Forward	5΄-CCTTCAACACCCCTGCCATG-3΄	58	298	298
Reverse	5΄-TGCTCGAAGTCCAGGGCC-3΄	57		
Amelogenin (AMEL)^d^				
Forward	5΄-TTCTCACCAGTACCCTTCCTA-3΄	55	395	458
Reverse	5΄-TCAGAGGCAGGTCAGGAAGCA-3΄	55		

^a^T = annealing temperature in PCR.

^b^Aasen & Medrano [7].

^c^This research.

^d^Zhu *et al*. [16].

#### The amelogenin gene (AMEL)

**Figure 4:**
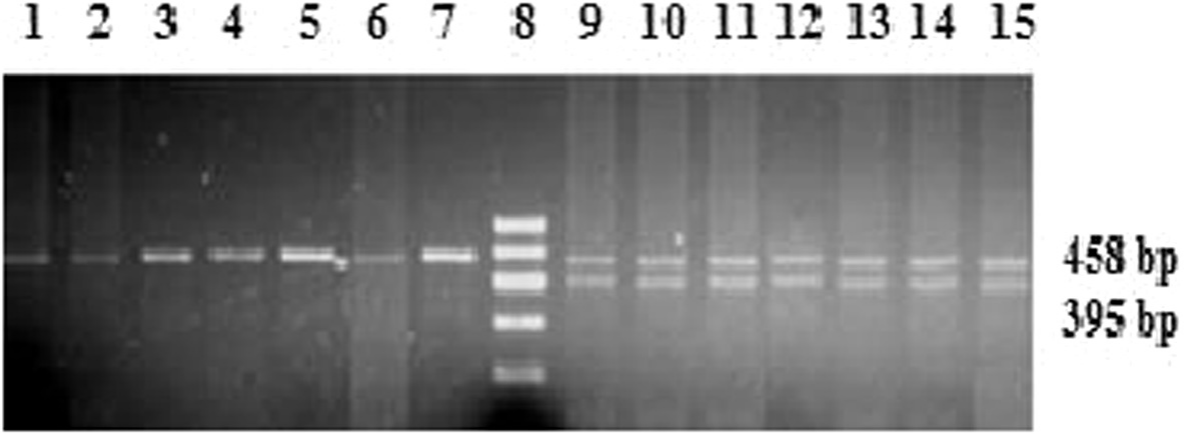
**Sexing of sheep known gender using PCR assays based on the amelogenin gene.** Male blood samples presented 458 bp and 395 bp bands while female blood samples had only 458 bp bands. Lanes 1–7 are PCR products of genomic DNA from seven female sheep; lanes 9–15 are PCR products of genomic DNA from seven male sheep; lane 8 is 600 bp DNA ladder.

After amplification with amelogenin primer, PCR product indicated a 458 bp and 395 bp fragments corresponding to the sheep blood genomic DNA. As expected, male samples presented both bands while female samples had only 458 bp band. For confirming the accuracy of the PCR, 14 blood samples (7 females and 7 males) were detected (Figure 4). All PCR sex determination was in agreement with the actual sexes of the sheep from which blood samples were obtained, indicating that the sexing method based PCR amplified amelogenin gene was 100% reproducible and reliable. 100% (14/14) concordance was obtained using the PCR assay.

#### The ZFY-ZFX gene

**Figure 5:**
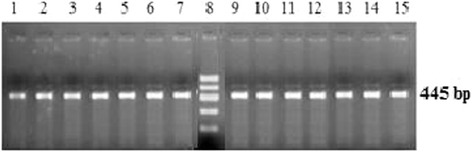
**Verification of the simple PCR products, using ZFY/ZFX-specific sequences primers.** The genomic DNA was extracted from male and female sheep. Lanes 1–7 are PCR products of genomic DNA from seven female sheep; lanes 9–15 are PCR products of genomic DNA from seven male sheep; lane 8 is 600 bp DNA ladder. The position of the 445 bp is the ZFY/ZFX product.

By choosing universal primers from sequences that are highly conserved in the X and Y chromosomes, sex-specific sequences were successfully amplified for genomic DNA. The results of the verification of the simple PCR products of genomic DNA extracted from male and female sheep, using ZFY/ZFX-specific sequences primers are shown in Figure 5.

### Sex determination

#### The HMG box

**Figure 6:**
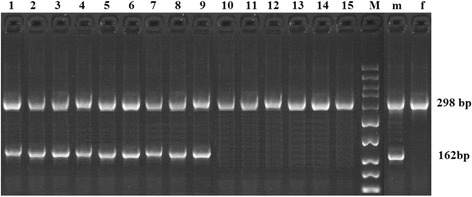
**The sex determination results of** ***ovine*** **primary satellite cells using the duplex PCR system.** Lane m and f are male and female blood samples, respectively; lane M is 50 bp DNA ladder; lanes 1–9 are male cells samples; lanes 10–15 are female cells samples; upper band (298 bp) corresponds to the positive control beta-actin product and it is present in all cells. Lower band (162 bp) corresponds to the HMG-box product and it is present only in males.

The sex identification results of primary satellite cells by duplex PCR were shown in Figure 6. The gel electrophoresis results displayed that the male-specific 162 bp fragments were obtained in male cells and only 298 bp internal control gene fragments were presented in female muscle satellite cells.

#### The amelogenin gene

**Figure 7:**
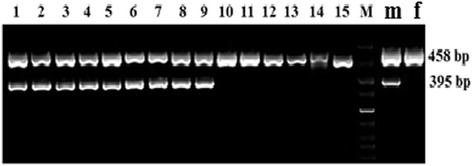
**The sex identification results of** ***ovine*** **primary satellite cells based on amelogenin gene.** Lane M is 50 bp DNA ladder; lane m and f are male and female blood samples, respectively; lanes 1–9 are PCR products of male cells samples; lanes 10–15 are PCR product of female cells samples.

The parts results of primary satellite cells PCR sexing after gel electrophoresis were shown in Figure 7. The samples showing 458 bp and 395 bp fragment were considered to be male muscle satellite cells, while those showing only 458 bp fragment were considered to be female muscle satellite cells. At the same time, blood DNA samples of known sex were used as positive control. Results showed that these primers were specific for rams. The overall amplification products obtained showed 100% accuracy.

#### Restriction fragment length polymorphism of the ZFY-ZFX gene

**Figure 8:**
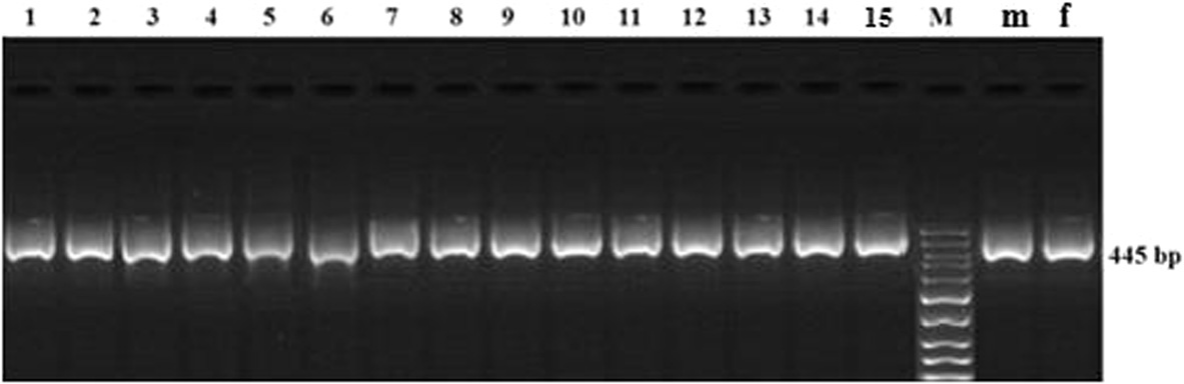
**Amplification of ZFY/ZFX-specific sequences using universal primers.** Lanes 1–15 are primary satellite cells samples; lane M is 50 bp DNA ladder; lane m and f are male and female blood samples, respectively. The cells subjected to PCR with universal primers showed uniform banding patterns (445 bp) irrespective of sex.

**Figure 9:**
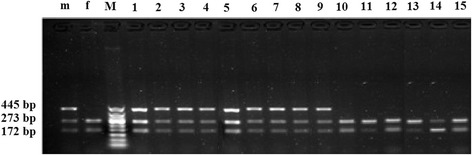
**Restriction patterns from** ***Sac*** **I digested ZFY/ZFX-PCR products of primary satellite cells samples.** Lane m and f are male and female blood samples, respectively; lane M is 50 bp DNA ladder; lanes 1–9 are PCR products of male cells samples; lanes 10–15 are PCR product of female cells samples. The ZFY fragment remained uncut while the ZFX homologue (which is present in both male and female cells) was digested to give fragments of 172 and 273 bp.

PCR with universal primers showed uniform banding patterns (445 bp) irrespective of sex (Figure 8) [7]. The sex was distinguished after digestion with *Sac*I, due to the presence of a single cut site (GAGCTC) on the sheep X chromosome which is absent on the Y-chromosome. Therefore, the ZFY fragment remained uncut while the ZFX homologue (which is present in both male and female cells) was digested to give fragments of 172 and 273 bp (Figure 9). The methods consistently produced fixed banding patterns in male and female DNA samples extracted from blood of known phenotypic sex. The overall sexing efficiency in the present study was 100%.

### Southern blot results

**Figure 10:**
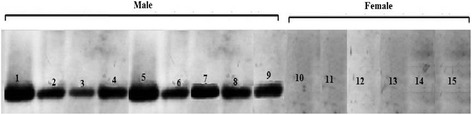
**Southern blot for sexing** ***ovine*** **primary satellite cells base on HMG box of SRY gene.** Lanes 1–9 are *ovine* genomic DNA of male cells samples (digested by *EcoRI* and *Hind*III). Lanes 10–15 are *ovine* genomic DNA of female cells samples (digested by *EcoR*I and *Hind*III).

To verify the specificity and accuracy of the amplification, southern blots were conducted with PCR derived products to confirm that the PCR products contained the HMG fragments. Southern blot results were in complete agreement with PCR-amplified bands (Figure 10). The HMG probe was hybridized with genomic DNAs from male and female *ovine*. The southern blot had only a single signal in Figure 8, which confirmed that the HMG box of SRY gene amplified from the *ovine* genome was specific for male.

## Discussion

In current study, we described mechanical and enzymatic disaggregation methods for the *in vitro* isolation and purification of sheep PSCs and found a comprehensive identification method for them. PSCs derived from enzymatic methods started isolation and growing from tissues faster than mechanical method. In contrast with the method of Gharaibeh *et al*. [10] (0.2% type IV collagenase digestion for 1 hr followed by 0.1% trypsin digestion for 30 min), the tissue was digested with type I collagenase that resulted in a more effective separation of cell from tissue. In accordance with our methods, Wu *et al*. [1] achieved better separation via digestion with 0.1% type I collagenase. Wu *et al*. [1] reported that type IV collagenase is less effective for cell separation than type I collagenase at the same concentration. Overall, the three-step digestion method, by increased frequency of digestion and decreased the incubation time, lead to increase the percentage of isolated cells and reduce the damage of cells.

The correct selection procedure is a key factor of successful gene targeting that lead to increase the cloning efficiency. Therefore, we used the GFP reporter vector to serve as selectable marker for gene targeted cell clones and also as a target vector to increase the transfection efficiency.

Many clones during the gene targeting procedure could not proliferate enough in normal medium because they have become senescent [11]. To increase cloning efficiency in our experiment, we used mitotically inactivated, non-multiplying “feeder” cells and bFGF because of continuous growth of PSC clones normally required bFGF and mitotically inactivated cells. In the current study, one method for simple and rapid plating of single satellite cells was described. In this procedure, “feeder” cells were used to condition the medium. Our results indicated that by extension of this method, it is possible to isolate transfected clones and grow pure clonal stocks of animal cells. We also showed that adding bFGF to growth medium could overcome the proliferation problem of fetal satellite cells during the single cell cloning (Table 1). The bFGF is a cell growth factor involved in angiogenesis and tissue repair. Previous research has demonstrated that bFGF suppresses cellular senescence in human mesenchymal stem cells [12] and *ovine* fetal myoblast cells [13].

Several approaches have been used to determine the sex of cells and embryos. Among of these methods, PCR is a rapid and easy procedure for large scale sexing and primers have been used to screen blood, cell and meat samples. In the present study, we compared several common approaches used in sex detection for the sexing of *ovine* primary satellite cells based on the ZFY/ZFX, amelogenin and high-motility-group (HMG) box using simple and duplex PCR. The sheep HMG box of SRY gene together with the beta-actin gene was simultaneously amplified as an internal control gene, which made the result more reliable. Results obtained by this method indicated that the method was sensitive enough to be utilized to sex the cells prior to produce sheep embryos via Somatic Cell Nuclear Transfer (SCNT) technique *in vitro*. Results showed that these primers were specific for rams. These observations were consistent with those reported by other researchers [14,15] for HMG box of goat and sheep. Shi *et al*. [15] showed that the male-specific 162 bp fragments were obtained in male embryos and only 298 bp internal control gene fragments were presented in female embryos. They demonstrated that the sex of the offspring was fully consistent with the results of PCR amplification and the accuracy of predictions was 100%.

Results of sex determination by using the amelogenin gene showed that these primers were specific for rams. This result was comparable to that reported by Zhu *et al*. [16] with the similar primers. This assay provides a rapid and accurate method for sexing, because of the presence of the X-chromosome band. Moreover, it can be carried out in a regular laboratory or under farm conditions within 3 hrs. This is especially important for the future application of the protocol to sheep cells sexing.

In the present study, universal primers were chosen from sequences that are conserved between human ZFY and ZFX genes and the mouse ZFY-1 and ZFY-2 genes. This locus is present on the Y chromosome of all placental sites [17,18]. Both ZFY and ZFX genes are phylogenetically conserved, so presence of restriction enzyme recognition sequence on this locus confers a polymorphism between the sexes. Amplification of ZFY and ZFX loci by PCR followed by detection of polymorphism between these amplified loci by RFLP has been used successfully in sex determination of many mammalian species [18,19]. These findings are in consonance with those reported by other authors [7,19].

The sex determination results based on HMG box by using the duplex PCR system need internal control gene, whereas, our findings showed that the PCR assay based on the amelogenin genes is fast and reliable for sex identification of sheep satellite cells. The advantage of this method was that neither additional control amplicons with a second locus specific autosomal primer pair nor restriction enzyme steps were necessary for sex determination and control of the PCR reaction. Moreover, it can be carried out in a regular laboratory or under farm conditions within 3 hrs. The PCR amplification of ZFY and ZFX gene followed by digestion with *Sac*I offer an accurate method of sex diagnosis of sheep satellite cells. However, this method is more time consuming and expensive than other methods.

## Conclusion

We successfully isolated and cultured sheep primary satellite cells via mechanical and enzymatic disaggregation. Two different types of proteases (type I collagenase and trypsin-EDTA) used to hydrolyze the primary satellite cells from muscle tissue. Our results indicated that enzymes treatment yielded the highest number of cells compared with mechanical disaggregation. Furthermore, our findings demonstrated that use of feeder and addition of bFGF to the culture medium improved cloning efficiency. Primary satellite cells generated in this experiment may be used as potential donor cells in somatic cell nuclear transfer programs. In addition, the results of sex detection demonstrated that the method consistently produced fixed banding patterns in male and female DNA samples extracted from blood of known phenotypic sex. Moreover, our results showed that the PCR assay based on the ZFY/ZFX, amelogenin and HMG box of SRY genes can apply for sex diagnosis of *ovine* satellite cells. Overall, these methods can be applied to detect the sex of primary satellite cells and to determine the sex of sheep embryo prior to produce sheep embryos by somatic cell nuclear transfer technique *in vitro*. Nevertheless, our findings suggested that sex determination of satellite cells based on amelogenin sequence can be accurate, relatively simple, rapid, and inexpensive.

## Methods

### Primary satellite cells generation

Muscle tissues were collected from 50 to 60-day-old sheep fetuses and sustained in Dulbecco’s Modified Eagle Medium (DMEM) (Gibco, Life Science, USA) containing gentamycin until use for mechanical and enzymatic disaggregation. Briefly, small tissue samples (1-mm cubes) were washed with DMEM two times and then transferred to a culture flask (~20–30 pieces per 25-cm^2^ flask) containing growth medium. The flask was placed in an incubator (Sanyo Electric Co., Ltd., Osaka, Japan) at 37°C until the cells were migrated from the tissues. The cell suspension was collected by centrifugation (5 min at 300 *g*) and then the cells cultured in growth media to initiate proliferation. The cells were plated in culture dishes coated with 0.1% gelatin (Sigma-Aldrich, Louis, USA).

For enzymatic digestion, the samples were digested in three steps with two different digestion enzymes including collagenase and trypsin-EDTA. Muscles tissue was digested with 0.1% type I collagenase (Sigma-Aldrich, Louis, USA) for 30 min, 0.25% trypsin (Gibco, USA) for 30 min following by repeat the digestion with 0.1% type I collagenase for 30 min at 37°C. After each digestion step, the hydrolyzed cells were filtrated through 40 μm mesh, and were then centrifuged (350 *g*, 20 min) at room temperature. After removal of the supernatant, the cells were seeded on 0.1% gelatin coated dishes and cultured in growth medium (GM): DMEM supplemented with 20% FBS, 10% horse serum, 50 mg ml^−1^ gentamycin at 37°C and 5% CO_2_. Sheep skeletal muscle satellite cells were purified using the differential adhesion method described by Gharaibeh *et al*. [10]. Identification of sheep PSC was done by RT-PCR reactions that were performed with Pax7 (forward, 5΄-GAGAAGAAAGCCAAGCACAGC-3΄, and reverse, 5΄-TACGCTTCAGAGGGAGGTCG-3΄), Desmin (forward, 5΄-AGCTGCTGGACTTCTCGCT-3΄, and reverse, 5΄-GCGAAGCGATCATTGAGC-3΄) and CD34 (forward, 5΄-ATGCTGGGCCGCAGGGGCGCG-3΄, and reverse, 5΄-GGTCTTCCGGGAATAGCTCTGGTG-3΄) primers. All experimental procedures were approved by the Biological Studies Animal Care and Use Committee, Beijing, Peoples Republic of China.

### Transfection of PSCs

The GFP reporter vector (pEGFP-N1, Invitrogen, CA, USA) was used for fast detection of positive clones in subsequent FACS analysis. Transfection was performed with the lipofectamine 2000 reagent (Invitrogen, CA, USA) combined with 5 μg of the pEGFP-N1 plasmid for first and second transfection time. Briefly, the cells were seed in 3 × 6-well plates and cultured until 80% confluence. The day before transfection, the medium was removed and 2 ml of fresh complete medium was added to cells prior to transfection. Experimental mixture was prepared by adding the following reagents: pEGFP-N1 plasmid, lipofectamine 2000 reagent and serum free medium (Life Technologies, USA) as recommended by the manufacturer. The mixture was incubated at room temperature for 5 min then added to the cells dropwise. In continuation, the cells were incubated in a CO_2_ incubator at 37°C. Then, three plates per treatment were collected for GFP expression assay as a following procedure: days 1 and 2 of first transfection continued with 3 days post-first transfection (day 5). Next 9 plates from treated cells were prepared for second transfection. Second transfection was done at day 5 of transfection for 48 hrs (day 7) as described above. Finally, three plates of cells were collected after 7 days and the rest were incubated 3 days post transfection for single cell cloning.

### FACS analysis of GFP expression and single cell sorting

The GFP expression was used for fast detection of integration-positive clones in subsequent FACS analysis. GFP expression in PSCs was photographed under a fluorescent microscope following days 1, 2 and 7 of transfection. Analysis of GFP expression was performed using the FACS (MoFlo®, Astrios™) system. For FACS analysis of GFP expression, the cells were collected on days 1, 2, 5 and 7 of transfection by centrifugation and washed in PBS.

GFP-positive single cell sorting from live second transfected pool was performed using FACS system in 20 × 96 well plates according to the index sorting function. Briefly, following 3 days post-second transfection the cells with a pEGFP-N1 vector, primary cells were detached by 0.25% trypsin-EDTA. The cell suspension was washed twice with PBS and cell pellet was resuspended in sorting buffer [PBS containing 0.2 mM EDTA and 1 mg ml^−1^ bovine serum albumin (BSA) at 1 × 10^6^ cells ml^−1^]. One cell was sorted into one well of a 96-well plate that contained growth medium. After sorting, all wells were checked for a single cell per well under an inverted microscope. The cultured transfected cells were single cell cloned afterward.

### Single-cell cloning

In this experiment, bFGF (basic Fibroblast Growth Factor) and sheep feeder cells were used to increase the cloning efficiency. In the first step, after single-cell sorting, sheep transfected PSCs were grown on 20 × 96-well plates coated with 0.1% gelatin until the first passage. The cells were cultured in GM and incubated at 37°C with 5% CO_2_. Half medium volumes were exchanged every 3 days for 14 days. In second step, the wells that contained cell clones were treated with 0.25% trypsin-EDTA and classified into 3 groups (Table 1), this clones were used for second cloning. The 288 clones were cultured in GM containing 20% FBS and other 288 clones were cultured in GM containing 20% FBS and 10 ng ml^−1^ bFGF onto 0.1% w/v gelatin-coated well plates (12 × 48) as group 1 and group 2, respectively. After 2 days, most of the medium was aspirated from the wells and it was replaced with fresh culture medium without bFGF. For all subsequent feedings, bFGF was not added to the growth medium. Five days after re-stimulation the clones in group 2, also for clones derived from group 1, the cells were harvested, centrifuged, and counted. The cells were re-suspended in fresh culture medium and cultured in 24-well plates. An aliquot of cells should be stimulated every week by bFGF. Cells generally live for 2–3 weeks after re-stimulation and should be frozen before they begin to die. For group 3, sheep feeder cells were grown on basic culture medium separately. For convenience these feeder cells should be prepared the day before the first passage. The feeder cells were plated at a density of 10^4^ cells per well onto 0.1% w/v gelatin-coated 48-well plates (6 × 48 wells). Next morning, sheep feeder cells were mitotically inactivated by treatment with mitomycin C (Sigma-Aldrich, Louis, USA) at a concentration of 10 μg ml^−1^ for 4 hrs at 37°C. Finally, the harvested cloned cells were cultured with confluent mitotically inactivated feeder cells in GM (2 ml per well).

### RNA isolation and RT-PCR analysis

Fifteen clones were chosen randomly between clones derived from single cell cloning. When the cells reached approximately 70% confluence, cells were collected for RNA extraction. Total RNA was isolated from the cells using TRIzol™ reagent (Invitrogen Co., Carlsbad, USA) based on the manufacture’s description. The amount of RNA was quantified using a spectrophotometer (ND-1000, Nanodrop Technologies Inc., Wilmington, USA) and the quality of RNA was evaluated by separation using agarose gel electrophoresis. The first strand of cDNA was synthesized using 1 μg of total RNA as a template, oligo-dT primer and SuperScript™ IIRNase H- Reverse Transcriptase (Invitrogen Co., Carlsbad, USA) according to the manufacturer’s instructions. Primers for PT- PCR analysis listed in Table 2.

### Condition of PCR and detection of amplified product

PCR conditions and PCR product sizes are shown in Table 2. In order to further shorten the amplification time, the thermal cycle parameters were optimized. PCR was programmed for 94°C for 3 min, followed by 35 cycles of one denaturation step at 94°C for 35 sec, primer annealing at 55-58°C (depending on the marker) for 60 sec and primer extension at 72°C for 120 sec. During the last cycle, the samples were incubated at 72°C for 10 min. Amplification products (10 μl) were analyzed on 2% agarose gel stained with golden view and evaluated under ultraviolet light.

### Blood samples preparation

Blood samples were collected from 7 males and 7 females as a control for further study. The blood samples were treated with anticoagulant before transport to the laboratory.

### DNA extraction from sheep blood

Genomic DNA samples isolated from the blood of known phenotypic sex were used as male and female controls for the verification of accuracy, sensitivity and specificity of PCR. Genomic DNA was extracted from blood by cell genomic DNA extraction kit (TIANGEN Biotech, Beijing, CO), according to the manufacturer’s instructions. The yield and purity of DNA samples were estimated by spectrophotometer (AstraNet, USA).

### Primer design for sex detection

For sex detection analysis, fifteen clones randomly selected between clones derived from single cell cloning. The primer sequences used in this study are listed in Table 2. The primers were designed with NTI advance™ 11 vector software (Life Technologies, Grand Island, NY, USA). The sheep male-specific primers and internal control primers were designed based on the HMG box of the sheep SRY gene sequence (GenBank Accession number: Z30265) and the sheep beta-actin mRNA sequence (GenBank Accession number: AF035422) for the duplex PCR. These primers for PCR amplification of a 162 bp region of HMG box sequence were developed. Moreover, primers for PCR amplification of a 447/445-bp region of the ZFY-ZFX genes were taken from Aasen & Medrano [7]. Amplification products were expected in males and females and were used as a positive control for successful PCR. This system was chosen as a control because of its known success in amplification of DNA from several mammals, including sheep. PCR products of the ZFY-ZFX genes (15 μl each) were subjected to digestion at 37°C for 3 hrs with 20 U of *Sac*I restriction enzyme (New England Biolabs, Ipswich) in the presence of 1× BSA (New England Biolabs, Ipswich) (2 mg), and then inactivated at 70°C for 10 min. The restriction fragment length polymorphism (RFLP) was analyzed using 2% gel electrophoresis and visualized under UV light [19]. Primers for PCR amplification of a 458/395-bp region of the amelogenin genes were taken from Zhu *et al*. [16]. The PCR product of female sheep sample is 458 bp long and male product is 395 bp long.

### Southern blot analysis

Male and female *ovine* genomic DNA was digested by two restriction enzymes, *EcoR*I and *Hind*III. The DNA was resolved on 1% (w/v) agarose gel and then transferred onto a positive charged nylon membrane with a vacuum-transfer system. The probe was prepared by PCR amplification using the forward primer 5΄-TGAACGCTTTCATTGTGTGGTC-3΄ and reverse primer 5΄-GCCAGTAGTCTCTGTGCCTCCT-3΄ designed based on the HMG box of the sheep SRY gene sequence (GenΒank Accession number: Z30265). Hybridization and detection were performed using a digoxigenin (DIG) high prime DNA labeling and detection starter kit II (Roche Molecular Biochemicals, Indianapolis, IN, USA) according to the manufacturer’s instructions.

## Abbreviations

DMEM: Dulbecco’s Modified Eagle Medium
FBS: Fetal bovine serum
FACS: Fluorescence-activated cell sorting
